# Supplementary material for: Alteration of actin cytoskeletal organisation in fetal akinesia deformation sequence
Source: Sci Rep. 2024 Jan 19;14:1742. doi: 10.1038/s41598-023-50615-1 (PMC10799014; doi:10.1038/s41598-023-50615-1)
Supplement: Supplementary file 1 — Supplementary Information. [file 41598_2023_50615_MOESM1_ESM.pdf]

Alteration of the actin cytoskeletal organisation in fetal akinesia deformation sequence

Ramona Jühlen, Lukas Grauer, Valérie Martinelli, Chantal Rencurel & Birthe Fahrenkrog

Supplementary information

Supplementary figures

Figure S1. Workflow of micropattern analysis. Each analysis step is numbered. (**1-7**) Immunofluorescence image stacks were automatically analysed using a macro written in ImageJ. Images were filtered, threshold was set and images centred. Images were filtered for single nuclei and interphase cells. Stacks were aligned using plugins MultiStackReg and TurboReg. Finally, a reference cell for each colour channel was created using Z-projections with a Rainbow RGB colour-coded Lookup Table. (**8-9**) Actin filament organisation (branches and junctions) as well as actin and focal adhesion orientation was investigated in ImageJ using plugins Analyze Skeleton and OrientationJ Analysis, respectively. (**10**) Actin filament and focal adhesion properties were analysed in detail by FilamentSensor[1]. (**11**) NMIIA organisation was analysed as described[2].

Figure S2. Non-muscle myosin peak frequency and the number of actin branches and junctions are unchanged in FADS fibroblasts. Violin plots of (**a**) NMIIA peak frequencies in actin arcs, (**b**) NMIIA peak frequencies in ventral stress fibres, (**c**) actin branches per cell, and (**d**) actin junctions per cell. NMIIA peak frequencies were analysed as described before[2]. Actin branches and junctions were measured in ImageJ using plugins Analyze Skeleton. n denotes the number of observations and μ the median (red dot). px, pixel.

Figure S3. Scratch-wound assays and levels of myosin phosphorylation in fibroblasts. Wound healing assays in MRC5 and FADS fibroblasts. Live fibroblasts were stained for 1 h with 62.5 nM SiR-actin, 4 h after wounding by a scratching with a 10 μl pipette tip. (**a**) Shown are representative bright-field (t=0 h) and SiR-actin time lapse images (t=5 h and t=29 h). The original wound area is outlined in turquoise in the bright-field images. Red

arrowheads in FADS 1 and FADS 2 indicate early migrating cells at t=5 h. Grey-values of SiR-actin were adjusted to MRC5 to underline over-saturation in FADS fibroblasts (t=29 h) and are presented as colour-coded Lookup Table. The colour calibration map indicates pixel intensities from 0 to 255. (**b**) Percentage of wound closure, (**c**) rate of cell migration (in µm/h), (**d**) mean wound area (µm^2) for culture inserts (Fig. 4c) and scratch wounds, and (**e**) mean wound width (in µm) for culture inserts (Fig. 4c) and scratch wounds (**a**) were calculated after image analysis using the ImageJ plugin Wound Healing Size Tool[3]. Dots are means of three and respectively five experiments for culture inserts and scratch wounds, vertical lines are SEM. Curves in (**b**) and (**c**) are fitted models of the raw data and curves in (**d**) and (**e**) are lines connecting the means. In (**d**) and (**e**) t=0 h were determined in bright-field images after wounding. Errors at some points are too small to be visible underneath the dots. (**f**) Shown are representative bright-field (t=0 h) and SiR-actin time lapse images (t=5 h and t=26 h) of scratch-wound assays in siRNA-treated MRC5 cells. The original wound area is outlined in turquoise in the bright-field images. Grey-values of SiR-actin are presented as colour-coded Lookup Table. The colour calibration map indicates pixel intensities from 0 to 255. (**g**) Percentage of wound closure, (**h**) migration rate (in µm/h) were calculated after image analysis using the ImageJ plugin Wound Healing Size Tool[3]. (**i**) Western blot analysis of the expression levels of non-muscle myosin heavy chain II-A (MHC) and phospho-myosin light chain (Ser19; MLC-P) in the indicated fibroblasts after wound healing assays and (**j**) their densitometric quantification. GAPDH was used as loading control. Complete blots are shown in Supplementary Fig. S6b online.

Figure S4. Negative controls for proximity ligation assays. PLAs were either conducted without primary antibodies (probes only) or with each primary antibody only (anti-rapsyn, anti-vinculin, anti-NUP88).

Figure S5. Bimolecular fluorescence complementation assays. Confocal microscopy analysis of BiFC signal produced by between (**a**) NUP88:actin and rapsyn:actin, (**b**) NUP88:vinculin and rapsyn:vinculin, and (**c**) NUP88:rapsyn in HeLa cells. The deletion Δ346-349 in actin modifies its hydrophobic binding cleft and the E167A and S350A mutations substitute amino acids surrounding the binding cleft. The D434Y and E634del mutations in NUP88 are FADS-related. DNA was stained with DAPI (blue). Shown are representative immunofluorescence images from at least three independent experiments. (**c**) Approximately 150 transfected cells were analysed per condition. ns, *p* not significant. Two-Way Anova test was used to calculate statistics.

Figure S6: Complete blots of blots shown in Fig. 4c and Supplementary Fig. S3f online.

References

1 Eltzner, B., Wollnik, C., Gottschlich, C., Huckemann, S. & Rehfeldt, F. The filament sensor for near real-time detection of cytoskeletal fiber structures. *PLoS One* **10**, e0126346 (2015). https://doi.org:10.1371/journal.pone.0126346

2 Hu, S. *et al.* Reciprocal regulation of actomyosin organization and contractility in nonmuscle cells by tropomyosins and alpha-actinins. *Mol Biol Cell* **30**, 2025-2036 (2019). https://doi.org:10.1091/mbc.E19-02-0082

3 Suarez-Arnedo, A. *et al.* An image J plugin for the high throughput image analysis of in vitro scratch wound healing assays. *PLoS One* **15**, e0232565 (2020). https://doi.org:10.1371/journal.pone.0232565

Table S1. Plasmids used in this study.

Table S2. Primers used in this study.

Figure S1

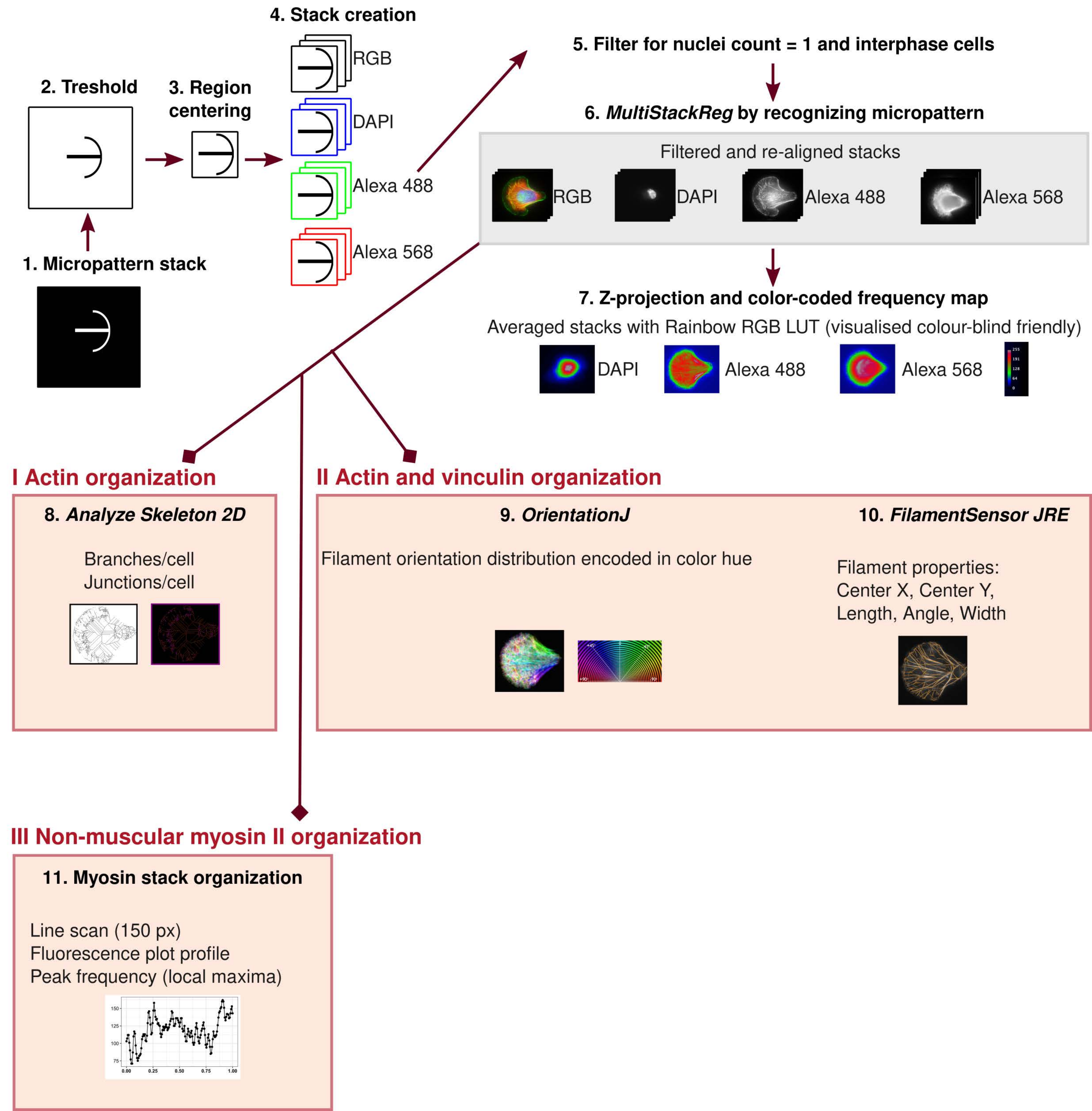

4. Stack creation
RGB
DAPI
Alexa 488
Alexa 568
2. Treshold
3. Region centering
1. Micropattern stack
5. Filter for nuclei count = 1 and interphase cells
6. MultiStackReg by recognizing micropattern
Filtered and re-aligned stacks
RGB
DAPI
Alexa 488
Alexa 568
7. Z-projection and color-coded frequency map
Averaged stacks with Rainbow RGB LUT (visualised colour-blind friendly)
DAPI
Alexa 488
Alexa 568
I Actin organization
8. Analyze Skeleton 2D
Branches/cell
Junctions/cell
II Actin and vinculin organization
9. OrientationJ
Filament orientation distribution encoded in color hue
10. FilamentSensor JRE
Filament properties:
Center X, Center Y,
Length, Angle, Width
III Non-muscular myosin II organization
11. Myosin stack organization
Line scan (150 px)
Fluorescence plot profile
Peak frequency (local maxima)
150
125
100
75
0.00
0.25
0.50
0.75
1.00

Figure S2

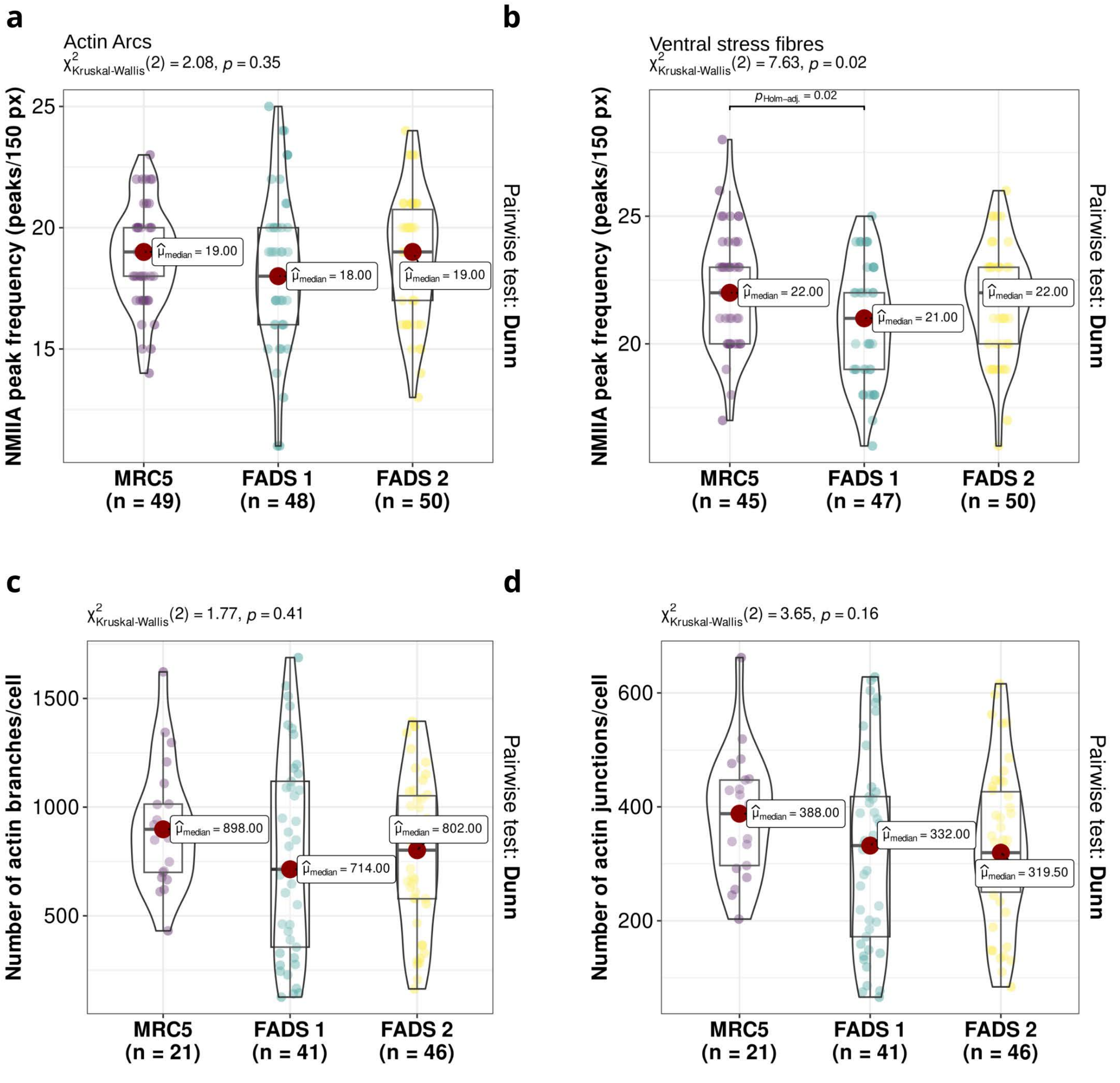
a
Actin Arcs
$\chi^2_{\text{Kruskal-Wallis}}(2) = 2.08,\ p = 0.35$
NMIIA peak frequency (peaks/150 px)
$\hat{\mu}_{\text{median}} = 19.00$
$\hat{\mu}_{\text{median}} = 18.00$
$\hat{\mu}_{\text{median}} = 19.00$
MRC5 (n = 49)
FADS 1 (n = 48)
FADS 2 (n = 50)
Pairwise test: Dunn
b
Ventral stress fibres
$\chi^2_{\text{Kruskal-Wallis}}(2) = 7.63,\ p = 0.02$
$p_{\text{Holm-adj.}} = 0.02$
NMIIA peak frequency (peaks/150 px)
$\hat{\mu}_{\text{median}} = 22.00$
$\hat{\mu}_{\text{median}} = 21.00$
$\hat{\mu}_{\text{median}} = 22.00$
MRC5 (n = 45)
FADS 1 (n = 47)
FADS 2 (n = 50)
Pairwise test: Dunn
c
$\chi^2_{\text{Kruskal-Wallis}}(2) = 1.77,\ p = 0.41$
Number of actin branches/cell
$\hat{\mu}_{\text{median}} = 898.00$
$\hat{\mu}_{\text{median}} = 714.00$
$\hat{\mu}_{\text{median}} = 802.00$
MRC5 (n = 21)
FADS 1 (n = 41)
FADS 2 (n = 46)
Pairwise test: Dunn
d
$\chi^2_{\text{Kruskal-Wallis}}(2) = 3.65,\ p = 0.16$
Number of actin junctions/cell
$\hat{\mu}_{\text{median}} = 388.00$
$\hat{\mu}_{\text{median}} = 332.00$
$\hat{\mu}_{\text{median}} = 319.50$
MRC5 (n = 21)
FADS 1 (n = 41)
FADS 2 (n = 46)
Pairwise test: Dunn

Figure S3

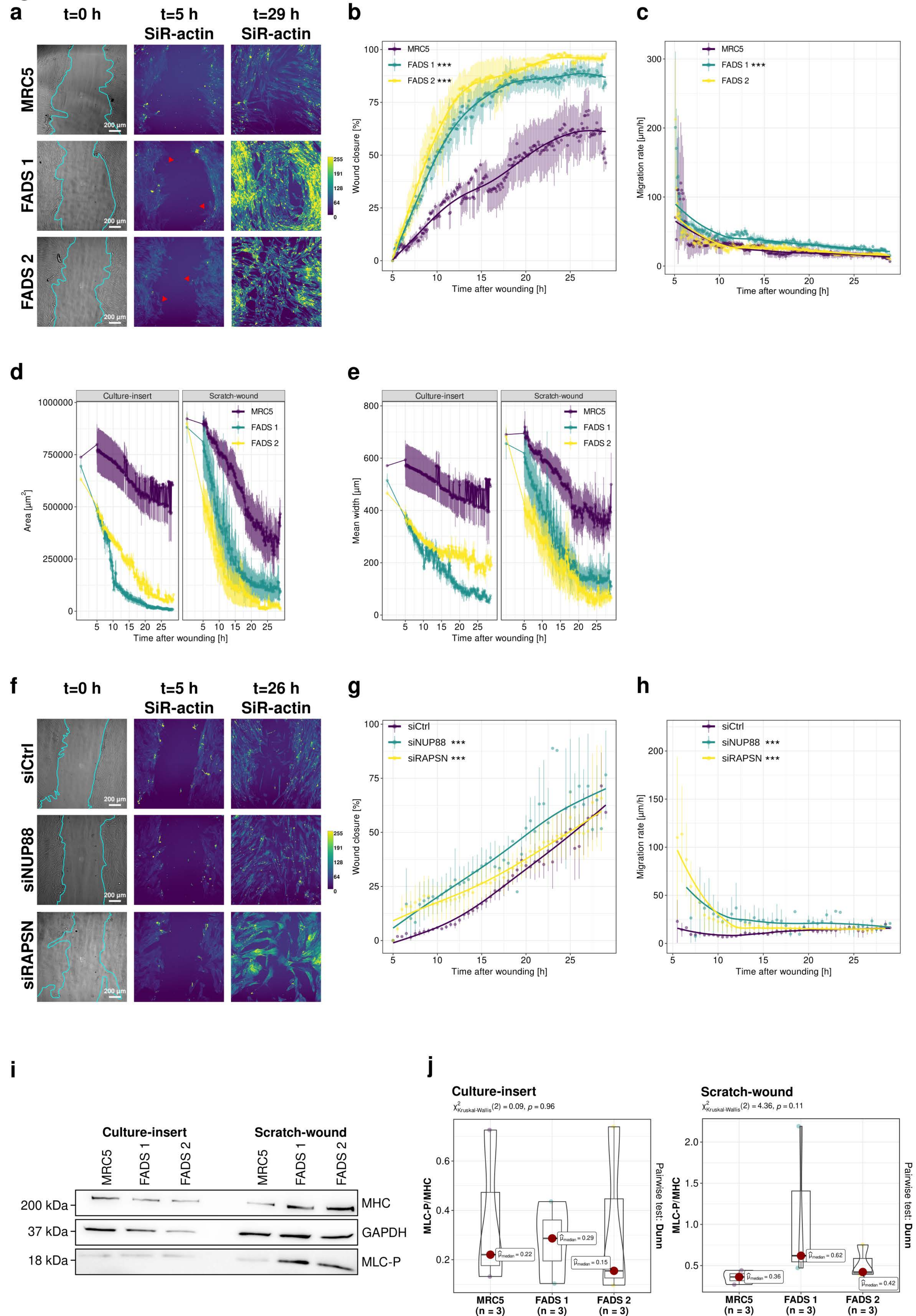

a
t=0 h
t=5 h SiR-actin
t=29 h SiR-actin
MRC5
FADS 1
FADS 2
200 µm
b
MRC5
FADS 1 ***
FADS 2 ***
Wound closure [%]
Time after wounding [h]
c
MRC5
FADS 1 ***
FADS 2
Migration rate [µm/h]
Time after wounding [h]
d
Culture-insert
Scratch-wound
MRC5
FADS 1
FADS 2
Area [µm2]
Time after wounding [h]
e
Culture-insert
Scratch-wound
Mean width [µm]
Time after wounding [h]
f
t=0 h
t=5 h SiR-actin
t=26 h SiR-actin
siCtrl
siNUP88
siRAPSN
g
siCtrl
siNUP88 ***
siRAPSN ***
Wound closure [%]
Time after wounding [h]
h
siCtrl
siNUP88 ***
siRAPSN ***
Migration rate [µm/h]
Time after wounding [h]
i
Culture-insert
Scratch-wound
MRC5
FADS 1
FADS 2
200 kDa
37 kDa
18 kDa
MHC
GAPDH
MLC-P
j
Culture-insert
χ2 Kruskal-Wallis(2) = 0.09, p = 0.96
MLC-P/MHC
μ̂ median = 0.22
μ̂ median = 0.29
μ̂ median = 0.15
MRC5 (n = 3)
FADS 1 (n = 3)
FADS 2 (n = 3)
Pairwise test: Dunn
Scratch-wound
χ2 Kruskal-Wallis(2) = 4.36, p = 0.11
μ̂ median = 0.36
μ̂ median = 0.62
μ̂ median = 0.42

Figure S4

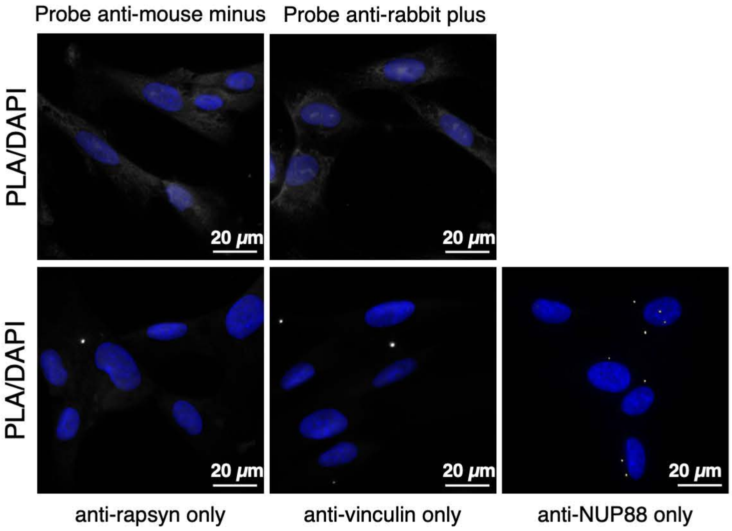

Probe anti-mouse minus
Probe anti-rabbit plus
PLA/DAPI
PLA/DAPI
20 μm
20 μm
20 μm
20 μm
20 μm
anti-rapsyn only
anti-vinculin only
anti-NUP88 only

Figure S5

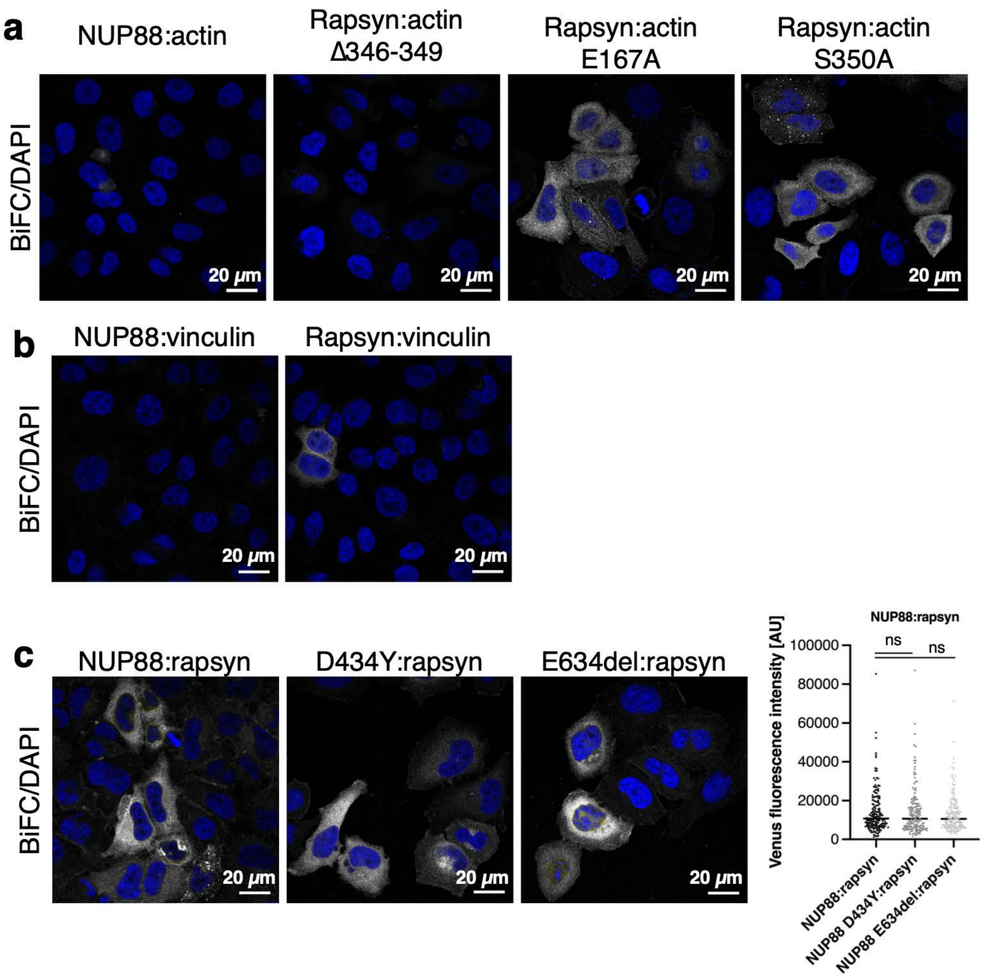

a
NUP88:actin
Rapsyn:actin Δ346-349
Rapsyn:actin E167A
Rapsyn:actin S350A
BiFC/DAPI
20 μm
b
NUP88:vinculin
Rapsyn:vinculin
BiFC/DAPI
20 μm
c
NUP88:rapsyn
D434Y:rapsyn
E634del:rapsyn
BiFC/DAPI
20 μm
NUP88:rapsyn
Venus fluorescence intensity [AU]
100000
80000
60000
40000
20000
0
ns
ns
NUP88:rapsyn
NUP88 D434Y:rapsyn
NUP88 E634del:rapsyn

Figure S6

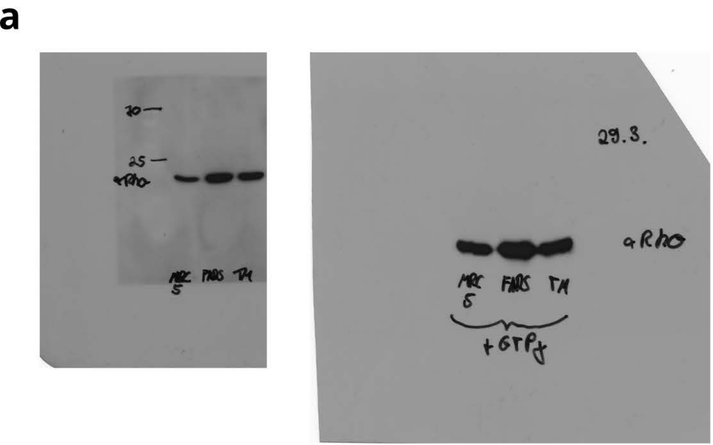

a
70
25
αRho
MRC 5
FARS
TM
29.3.
αRho
MRC 5
FARS
TM
+GTPγ

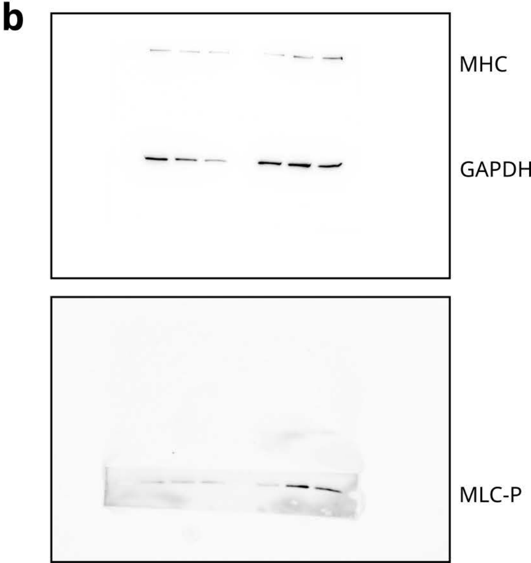

b
MHC
GAPDH
MLC-P

Supplementary Table 1: Plasmids used in this study

Plasmid	Construct	Source
PBF992	pDEST-ORF-V1	Addgene #73637
PBF993	pDEST-ORF-V2	Addgene #73638
PBF1035	pDEST-rapsyn-V1	Jühlen et al., 2020
PRL491	pDEST-vinculin-V2	This study
PRL492	pDEST-actin-V2	This study
PRL513	pDEST-NUP88-V1	This study
PRL556	pDEST-NUP88 D434Y-V1	This study
PRL577	pDEST-NUP88 E634del-V1	This study
PRL579	pDEST-rapsyn E162K-V1	This study
PRL581	pDEST-actin G521D-V2	This study
PRL635	pDEST-paxillin-V2	This study
PRL638	pDEST-actin Δ143-146-V2	This study
PRL639	pDEST-actin Δ346-349-V2	This study
PRL661	pDEST-actin E167A-V2	This study
PRL662	pDEST-actin S350A-V2	This study

Supplementary Table S2: Primers used in this study

**Target**	**Sense**	**Sequence**
pDEST-ORF-V1/V2	FWD	AACCCAGCTTTcttgtacaaagtggtcatc
	REV	TAAGCCTGCTTTTTTGTACaaacttgtctc
NUP88-V1	FWD	cAAAAAAGCAGGCTTAatgGCGGCCGCCGAGGgaccggtg
	REV	caagAAAGCTGGGTTGAAGTTTACATGATTGCGGATATCATTG
NUP88 D434Y-V1	FWD	CTTGGATCAGATGAAGAAtATAAGGATAGTTTACAGGAACTC
	REV	GAGTTCCTGTAAACTATCCTTATaTTCTTCATCTGATCCAAG
NUP88 E634del-V1	FWD	cAAAAAAGCAGGCTTAatgGCGGCCGCCGAGGgaccggtg
	REV	caagAAAGCTGGGTTGAAGTTTACATGATTGCGGATATCATTG
Rapsyn-E162K-V1	FWD	caatgatgacgccatgctcAagtgccgcgtgtgctgcagcctg
	REV	caggctgcagcacacgcggcactTgagcatggcgtcatcattg
Actin-V2	FWD	AAAGCAGGCTTAatggatgatgatatcg
	REV	caagAAAGCTGGGTTgaagcatttgcggt
Actin G251D-V2	FWD	gacggccaggtcatcaccattgAcaatgagcggttccgctg
	REV	cagcggaaccgctcattgTcaatggtgatgacctggccgtc
Actin E167A-V2	FWD	cactgtgcccatctacgcggggtatgccctcccccatg
	REV	catgggggagggcataccccgcgtagatgggcacagtg
Actin S350A-V2	FWD	gctccatcctggcctcgctggccaccttccagcagatgtg
	REV	cacatctgctggaaggtggccagcgaggccaggatggagc
Actin Δ143-146-V2	FWD	gtgctatccctgtacaccactggcatcgtgatggac
	REV	gtacagggatagcacagcctgg
Actin Δ346-349-V2	FWD	ggcggctccatcctgaccttccagcagatgtggatc
	REV	caggatggagccgccgatccac
Paxillin-V2	FWD	AAAGCAGGCTTAatggacgacctcgacgc
	REV	caagAAAGCTGGGTTgcagaagagcttgaggaagcag
Vinculin-V2	FWD	GTACAAAAAAGCAGGCTTAatGCCAGTGTTTCATACGCG
	REV	caagAAAGCTGGGTTCTGGTACCAGGGAGTCT
